# Large-Scale Drift-Resilient Localization via Multi-Sensor Fusion and Topological Map Matching

**DOI:** 10.3390/s26113495

**Published:** 2026-06-01

**Authors:** Xiaochun Yang, Chenxi Shao, Pengju Hou, Jie Yan, Wenxing Fu

**Affiliations:** 1 School of Astronautics, Northwestern Polytechnical University, Xi’an 710129, China; 2 School of Automation, Northwestern Polytechnical University, Xi’an 710129, China; 3 Xi’an Aerospace Saineng Automation Technology Co., Ltd., Xi’an 710100, China

**Keywords:** large-scale, robot localization, topological map matching, drift correction, HMM, odometry

## Abstract

**Highlights:**

**What are the main findings?**

- A localization framework based on multi-sensor fusion odometry and topological map matching.
- A robust multi-sensor fusion odometry with ground points filtering and a back-end factor graph that fuses multiple sensor measurements.
- A large-scale drift correction method that aligns historical trajectories with topological map through map matching.

**What are the implications of the main findings?**

- The proposed localization framework enables drift-resilient localization in large-scale road environments without the need for constructing high-precision environmental maps.
- The proposed odometry effectively filters out interference from ground points and tightly integrates data from multiple sensors, significantly improving both localization accuracy and robustness, particularly for ground-based robot platforms.
- The proposed drift correction method facilitates the estimation and correction of accumulated localization drift in large-scale road environments, without relying on pre-built high-precision maps, and only utilizes GNSS during initialization.

**Abstract:**

In large-scale road environments, constructing and maintaining high-precision maps is challenging, while GNSS-denied conditions exacerbate accumulated drift due to the lack of global references. Additionally, existing methods largely rely on LiDAR data but inadequately preprocess the data, which often leads to degraded accuracy and instability. To address these issues, this study proposes large-scale drift-resilient localization via multi-sensor fusion and topological map matching. The method leverages digital maps to extract topological road networks, eliminating the need for high-precision map construction. Accumulated drift is corrected by matching the odometry trajectory with the topological map, while localization accuracy and stability are further improved through precise ground point filtering and the integration of wheel odometry into a LiDAR-inertial odometry. Experiments on two campus datasets and KITTI 05 demonstrate the high accuracy and generalization of the proposed method in large-scale localization. Notably, on the longer School Dataset (3645 m), the mean error drops by 48.1% relative to LIO-SAM and 44.2% relative to FAST-LIO2. Repeated ablation trials further confirm the stability of the proposed method. These results demonstrate accurate and stable large-scale localization without high-precision prior maps.

## 1. Introduction

Autonomous navigation is essential for mobile robots, enabling them to perform tasks in diverse environments [1], especially in large-scale road environments like university campuses, industrial parks, and residential communities. Autonomous navigation technologies are increasingly empowering robots to perform tasks such as security patrols and logistics transportation in these environments [2].

A critical aspect of autonomous navigation is localization, which enables robots to determine their position and effectively avoid obstacles. GNSS can provide direct localization. However, in some outdoor environments, GNSS signals are often weak or blocked by tall buildings, trees or other obstructions, and high-precision GNSS systems remain cost-prohibitive. Given these limitations and owing to its stability and adaptability, LiDAR-based localization has become a widely adopted alternative [3]. However, in large-scale road environments, the wider coverage, increased complexity, and dynamic changes still pose significant challenges for LiDAR-based localization [4].

The first challenge is constructing and maintaining high-precision maps over vast areas, which is a complex and resource-intensive task. Some proposed localization methods use pre-built maps to match real-time LiDAR data, allowing the robot to estimate its position on the map while reducing drift [5]. However, constructing and maintaining maps in large-scale road environments remains challenging [6]. In these environments, the map construction process is time-consuming and large-scale, and without GNSS correction or loop closure detection, map building is highly susceptible to deviations [7]. Additionally, reference [8] indicates that high-precision LiDAR can generate point clouds at over one million points per second in urban road settings. As a consequence of such high acquisition rates, the accumulated data per square kilometer easily reaches hundreds of millions of points. Reference [9] further shows that even a map covering a space of 600 m^3^ already contains more than 17 million points and occupies nearly 1 GB of storage; high-definition maps for large-scale road environments will therefore inevitably require tens of gigabytes or more, imposing considerable pressure on data storage and transmission. Furthermore, continuous changes in buildings, roads, and obstacles cause maps to rapidly become outdated. As stated in reference [10], the map must undergo large-scale updates every 3 to 6 months, incurring high costs and demanding substantial computational resources.

The second challenge is accumulated drift. Some localization methods do not rely on pre-built maps but instead use historical point cloud data and real-time point cloud registration to construct LiDAR odometry for localization [11]. While these methods avoid the problem of constructing large-scale maps, they still face the common issue of accumulated drift in large-scale road environments. In such environments, long-distance driving can cause small errors between odometry frames due to sensor noise and point cloud registration errors, which accumulate over time, ultimately leading to large errors that compromise localization accuracy or even cause failure [12]. For instance, the LeGO-LOAM algorithm [13], a representative of pure LiDAR odometry, only loosely couples IMU data for motion distortion correction and result constraints, which causes larger frame-to-frame errors during significant attitude changes. In long-distance localization, this accumulated error grows substantially. On the public KITTI 05 benchmark, LeGO-LOAM accumulated an average error of 27.603 m and a maximum error of 67.561 m over a 2.2 km trajectory without loop closure. LIO-SAM [14], a representative of LiDAR-inertial odometry, tightly couples the IMU, leading to improved localization accuracy compared to LeGO-LOAM. However, it still cannot eliminate small frame-to-frame errors, which accumulate into larger errors over long distances. After 1219 m of testing, the error still accumulated to around 5 m. Despite incorporating GPS factors and loop closure detection to reduce drift, LIO-SAM faces challenges in large-scale road environments. Tall roadside buildings, bridges, and tunnels can block GNSS signals, and loop closure detection requires the robot to follow a specific route and revisit previous positions, which significantly impacts navigation efficiency. Existing methods to eliminate accumulated drift thus remain considerably limited.

Additionally, existing LiDAR-based localization methods largely rely on point cloud data, but most of these methods inadequately preprocess the data, leading to localization errors or anomalies. In road environments, when ground robots use LiDAR for localization, point cloud data often contains a large number of ground points, which lack effective features and are mixed with sensor noise, resulting in incorrect feature extraction and point cloud misalignment, thereby increasing localization errors and causing jumps in positioning [15]. While algorithms like LeGO-LOAM [13] optimize for ground robots by filtering ground points, they rely solely on angular changes between point clouds to roughly segment ground points, which often leads to misclassification and incorrectly filtering out non-ground planar points. Furthermore, these methods have poor adaptability in environments with significant slope changes. Ground robot localization in large-scale road environments therefore requires targeted optimization.

To address the challenges of map construction and accumulated drift, researchers have turned to open-source digital maps such as OpenStreetMap (OSM) [16] as an alternative source of global reference. Unlike high-precision prior maps, OSM provides globally available, continuously updated road network topology at no construction cost. Several recent works have leveraged OSM data for robot localization, demonstrating that digital maps can serve as effective priors for drift reduction [17,18]. However, existing digital-map-based methods still face notable limitations. They often focus on specific structured environments and have rarely been validated in large-scale road scenarios. Consequently, achieving robust, real-time localization in large-scale road environments using digital maps remains an open challenge.

In this study, to address the aforementioned issues, we propose a localization method called Large-scale Drift-Resilient Localization via Multi-Sensor Fusion and Topological Map Matching. Building on the promise of OSM as a freely available prior, our method extracts topological road networks from OSM to provide global reference information without requiring high-precision environmental maps. For the accumulated drift issue, we propose a topology-based map matching approach for drift correction. By extracting road network topological maps from OSM and matching them with the robot’s historical trajectory, we construct a Hidden Markov Model (HMM) [19] and apply the Viterbi algorithm [20] to estimate and eliminate accumulated drift without GNSS. Finally, to address the over-reliance on LiDAR and insufficient point cloud preprocessing, we use the efficient Line-Fit algorithm [21] to filter out ground points, significantly reducing their impact. LiDAR odometry is then fused with IMU pre-integration factors and wheel odometry, which directly measures the robot’s velocity via encoders on the chassis [22], in a back-end factor graph optimization to achieve multi-sensor fusion odometry construction. Ultimately, this design yields a drift-resilient localization system that requires no high-precision prior map and relies on GNSS only for initialization, making it particularly suitable for large-scale road environments. The main contributions of this study are as follows:

- A localization method based on multi-sensor fusion odometry and topological map matching, which provides localization through multi-sensor fusion odometry and uses topological map matching to eliminate accumulated drift, offering drift-resilient localization without requiring high-precision environmental maps.
- A topology-based drift correction method that estimates and eliminates accumulated drift without GNSS by matching the robot’s trajectory with a topological road network extracted from digital maps, using an HMM and the Viterbi algorithm for error correction.
- A multi-sensor fusion odometry system that accurately filters out ground point interference using the Line-Fit algorithm and integrates wheel odometry velocity factors into back-end factor graph optimization for higher precision localization.

The remainder of this manuscript is organized as follows: Section 2 reviews related work on LiDAR-based localization methods. Section 3 outlines the system framework of our methods. Section 4 details the tightly coupled multi-sensor fusion odometry. Section 5 presents the topology-based map matching approach for drift correction. Section 6 discusses large-scale road localization experiments conducted on a custom-built robotic platform. Section 7 gives the conclusion and further discussion.

## 2. Related Work

This study focuses on LiDAR-based localization in large-scale road environments. These environments typically refer to areas that extend over several kilometers and include scenes within 100 m of both sides of the road [23]. This section therefore provides a comprehensive review and analysis of the relevant literature on LiDAR-based localization. LiDAR-based localization methods can be broadly categorized into two main branches, map-based and map-less localization, depending on whether a pre-built map is used.

### 2.1. Map-Based LiDAR Localization

Map-based LiDAR localization requires a pre-built environmental map, which can be based on identifiable landmarks, such as high-reflectivity objects or pole-like features [24]. For instance, Xie et al. [25] used high-reflectivity objects to match with pre-labeled landmarks on a map to estimate the robot’s position. These methods, however, demand densely distributed landmarks in the environment for stable localization, which becomes more challenging in large-scale road settings due to the increased complexity of the area. Another common approach uses Simultaneous Localization and Mapping (SLAM) [26] to build high-precision 3D maps. These methods offer better adaptability and accuracy with minimal drift in complex environments. Algorithms like ICP and NDT register real-time point clouds with environmental maps [27,28], but they are computationally expensive and sensitive to initial conditions, often leading to local minima. To improve efficiency, some methods extract distinctive, sparser features from the map for matching, such as SC-LIO-SAM [29], which uses Scan-Context descriptors to reduce computational load while maintaining high accuracy.

In summary, although current research has explored various environmental maps and matching techniques, these methods all rely on constructing prior maps to achieve optimal localization performance. However, in the large-scale road environments addressed in this study, these methods must contend with larger areas and more complex environments. This makes the placement of landmark-based maps more difficult and requires the construction of massive, high-precision 3D point cloud maps, which are both challenging and resource-intensive to maintain.

### 2.2. Map-Less LiDAR Localization

Map-less LiDAR localization methods do not require a pre-built environmental map. By using only a single LiDAR sensor, these methods construct a LiDAR odometry system for localization through point cloud registration. Classic algorithms such as ICP and NDT can also be applied to map-less localization by matching consecutive real-time point clouds [30,31]. However, these methods are computationally expensive and highly sensitive to initial conditions, leading to suboptimal real-time performance and stability.

To address these problems, feature-based methods have been proposed. One of the most prominent approaches is the LOAM series of algorithms [11], which extract geometric features from point clouds and perform matching based on sparser and more representative feature points, improving computational efficiency while maintaining localization accuracy. LeGO-LOAM [13], a widely used ground LiDAR odometry system, has been optimized for ground vehicles by filtering out ground points and applying lightweight graph optimization, reducing computational burden and making it suitable for edge platforms. However, LeGO-LOAM’s loose coupling with the IMU and imprecise ground point filtering leads to suboptimal performance in large-scale road environments. Additionally, all single-sensor methods suffer from stability issues [32]. When the point cloud data captured by LiDAR is sparse, point cloud registration becomes unstable, and pose optimization can easily diverge. In environments such as long corridors or tunnels, where LiDAR performance degrades, the similarity between consecutive point clouds can be so high that pose optimization becomes trapped in local minima.

To enhance the stability and accuracy of LiDAR-based localization, sensor fusion techniques are therefore employed. The IMU, with its resistance to external environmental factors and high-frequency measurements, has become the industry standard sensor for fusion with LiDAR. Ye et al. [33] proposed LIO-Mapping, one of the earliest open-source LiDAR-inertial odometry methods, which jointly optimizes LiDAR point cloud matching and IMU measurements in a continuous-time framework, laying the foundation for subsequent LiDAR-inertial tight-coupling frameworks. Shan et al. [14] introduced LIO-SAM, which has become a base-line method for many open-source datasets. LIO-SAM uses a factor graph-based tight-coupling framework, integrating LiDAR odometry factors and IMU pre-integration factors for optimization, achieving high-precision, real-time robot localization. Additionally, it incorporates GPS factors and loop closure detection, which help correct accumulated drift when GPS data is available or when revisiting previous locations after a short time. The FAST-LIO series algorithms [34], proposed by Xu et al., use an ESIKF filter to optimize the localization result, representing a lightweight LiDAR-inertial odometry solution that offers high computational efficiency and stability. However, these methods do not effectively address the accumulated drift issues. Although some methods incorporate GPS or loop closure detection for drift correction, challenges remain in large-scale road environments. GNSS signals are susceptible to interference and can even fail. In GNSS-denied scenarios, methods relying on GPS or loop closure detection will be ineffective unless the robot follows a specific route to revisit previous positions [4].

In summary, map-less LiDAR localization methods have made significant progress but still face challenges in handling accumulated drift, especially in large-scale road environments. The reliance on GNSS and loop closure detection for drift correction is limited in scenarios where GNSS signals are unavailable or degraded. Additionally, while LeGO-LOAM has been optimized for ground vehicles, its ground filtering is not ideal, and its stability is inferior to multi-sensor fusion methods. Other methods have seen limited optimization for ground robots.

### 2.3. Localization via Digital Map and HMM

Recently, open-source digital maps like OpenStreetMap (OSM) have shown promise in addressing autonomous navigation challenges. OSM not only provides global guidance for robot path planning [35], but its location information also supports localization by offering reference points for the robot’s position.

For example, Frosi et al. [17] proposed OSM-SLAM, which integrates 2D building geometry from OSM into a LiDAR-based Graph SLAM system, improving localization accuracy and enabling re-localization when sensor data is lost. Li et al. [18] proposed a LiDAR-OSM matching method using building boundary features for global position initialization, aligning OSM contours with LiDAR-perceived features in the absence of prior LiDAR maps. While it outperforms traditional descriptors in urban areas, its accuracy depends on building boundary visibility, which may fail in open spaces. For map enhancement, Leitenstern et al. [36] introduced the FlexMap Fusion system, which integrates OSM updates into high-precision vector maps, reducing manual annotation costs. However, its real-time performance remains unverified. Ai et al. [37] integrated OSM-derived structural information into a factor graph optimization framework, combining LiDAR-inertial odometry with map-aided distance constraints. This approach suppresses cumulative drift in structured corridors by adding OSM-based constraints to the factor graph alongside LiDAR and IMU factors. Nevertheless, its reliance on the quality and density of OSM data, as well as its limited validation beyond structured environments, leaves its applicability in large-scale road scenes an open question.

Beyond the above OSM-based methods, topological map matching using Hidden Markov Models (HMM) has emerged as an effective probabilistic framework for robot localization, particularly for drift correction in large-scale environments. Li et al. [38] proposed the Roadnetwork-Constraint Hidden Markov Model (RC-HMM), which incorporates road network constraints into a topometric map to enhance localization accuracy. In a related direction, an HMM-based multi-frame LiDAR descriptor matching method was proposed in [39] for place recognition.

In summary, current LiDAR localization methods using open-source digital maps often focus on specific tasks or scenarios, relying on manual features and prior data quality. While recent HMM-based topological matching approaches show promise in reducing cumulative drift, their application to large-scale road environments remains limited. Expanding these methods to larger road environments and achieving robust, real-time localization remains an open research challenge.

## 3. System Framework

**Figure 1:**
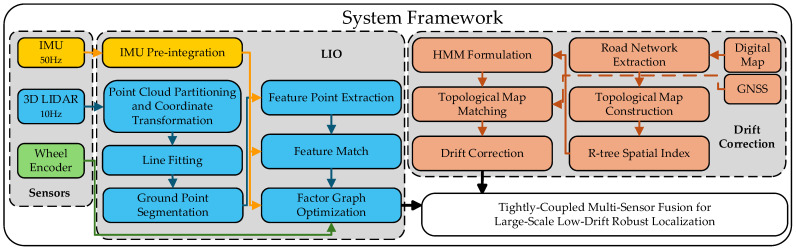
The overall system of our localization scheme, which fuses multi-sensor data. After ground point removal, a tightly coupled multi-sensor fusion odometry is built, and cumulative drift is corrected using a digital map, producing stable, low-drift poses. GNSS is only used for initial alignment, denoted by a dashed line.

The system framework of the proposed method is shown in Figure 1 and consists of two main components: multi-sensor fusion odometry and drift correction. The initial localization results are obtained from multi-sensor fusion odometry, and the drift correction component eliminates the accumulated drift, ultimately providing a drift-resilient localization solution.

The multi-sensor fusion odometry system takes IMU, LiDAR, and wheel encoder data as input sensors. The IMU pre-integrated results are used for motion distortion correction of the LiDAR and also serve as prior pose estimates for feature point matching. The IMU data ultimately acts as an IMU factor in the factor graph optimization. As the primary sensor, LiDAR first processes the input point cloud through partitioning and coordinate transformation. The Line-Fit algorithm is then applied to fit lines that describe the ground points, enabling ground point cloud segmentation and removal. After removing the ground points, feature point extraction and matching are performed, which leads to estimating the relative transformation between consecutive LiDAR frames to build the LiDAR odometry. The odometry results are introduced as a LiDAR factor into the factor graph. Finally, the velocity factor obtained from the wheel encoder measurements is added to the factor graph, followed by unified factor graph optimization to yield the final multi-sensor fusion odometry localization data.

The digital map is processed through road network extraction, topological map creation, and R-tree [40] spatial indexing. Afterward, the topological map and odometry trajectory are input into the drift correction component. An HMM is constructed, and accumulated drift is estimated and corrected via topological map matching. In this process, GNSS is only used for initialization to determine the robot’s position within the topological road network at system startup. Following these steps, precise, stable, and low-drift localization data is achieved.

To validate the effectiveness of the proposed multi-sensor fusion odometry and drift correction methods, a series of experiments were conducted and analyzed (Section 6). In addition to the public KITTI benchmark, real-world validation was performed on a self-built unmanned ground vehicle (UGV) platform. The sensor suite consisted of an LSLiDAR C32 LiDAR (Shenzhen Leishen Intelligent System Co., Ltd., Shenzhen, China) with 32 channels, a 360° horizontal field of view, a vertical field of view ranging from −16° to +15°, and a point cloud output frequency of 10 Hz, along with a TL740D MEMS IMU (Shenzhen Rion Technology Co., Ltd., Shenzhen, China). The ground-truth trajectory was provided by a CGI410 high-precision GNSS (Shanghai Huace Navigation Technology Ltd., Shanghai, China).

To evaluate the performance of the proposed method in large-scale road environments, two datasets were collected in different campus settings: the College Dataset and the School Dataset, with total travel distances of 1219 m and 3564 m, respectively. The robot platform has a maximum linear velocity of 1.5 m/s and a maximum angular velocity of 0.5 rad/s. All algorithms were implemented in C++ and Python 2.7 using the Robot Operating System (ROS) Melodic version and executed on a laptop equipped with an Intel i7-10750H CPU.

## 4. Multi-Sensor Fusion Odometry

To address the challenges of low accuracy and insufficient stability in road environment localization identified in prior work, this section introduces a multi-sensor fusion odometry system. Unlike LeGO-LOAM, which ground point filtering is unstable, our method incorporates a Line-Fit algorithm to more accurately filter out ground points, improving robustness in complex environments.

The proposed system constructs a LiDAR-inertial odometry pipeline using the ground-removed point cloud, similar to traditional LiDAR-inertial odometry, but extends it by integrating wheel odometry constraints within a factor graph optimization framework. This integration provides additional velocity constraints, which further improve localization stability, especially in large-scale environments.

### 4.1. Ground Point Cloud Filtering

Feature-based registration algorithms are designed for point cloud matching, so extracting features from ground points is problematic as these features lack distinctiveness, and stable correspondences cannot be reliably established. Efficient and accurate segmentation of ground points is therefore a critical preprocessing step. Since LeGO-LOAM employs a simple angle-based ground filtering strategy, it has limited adaptability to environments with significant slope variations and may erroneously classify planar surfaces of non-ground objects as ground points. In this work, we employ the Line-Fit algorithm [21], a stable and computationally efficient method for ground point segmentation, which proceeds through three main steps: point cloud partitioning and coordinate transformation, line fitting, ground point segmentation.

The first step involves dividing the point cloud into partitions and coordinate transformation. For a given point cloud set $P_{t}=\{p_{t}^{1},p_{t}^{2},\cdots ,p_{t}^{m},p_{t}^{m+1},\cdots ,p_{t}^{n}\}$, each point $p_{t}^{i}={\left(x_{t}^{i},y_{t}^{i},z_{t}^{i}\right)}^{T}$ is projected onto the XOY plane by discarding its Z-coordinate. A circular region centered at the LiDAR origin, with an infinitely large radius, is divided into N sectors $S_{s}(s=1,2,\cdots ,N)$, each spanning an angle of Δα=2π/N. Within each sector, the radial range between the minimum detection radius $r_{\min}$ and the maximum radius $r_{\max}$ is uniformly divided into $n_{bins}$ sub-sectors $b_{s,j}(j=1,2,\cdots n_{bins})$. Each 3D point $p_{t}^{i}\in P_{s,j}$ inside a sub-sector is transformed into a 2D representation ${p_{t}^{i}}^{\prime }\in {P^{\prime }}_{s,j}$, preserving its Z-coordinate and radial distance:

(1) $${P^{\prime }}_{s,j}=\{(r_{t}^{i},z_{t}^{i})|p_{t}^{i}\in P_{s,j}\},r_{t}^{i}=\sqrt{{\left(x_{t}^{i}\right)}^{2}+{\left(y_{t}^{i}\right)}^{2}}$$

**Figure 2:**
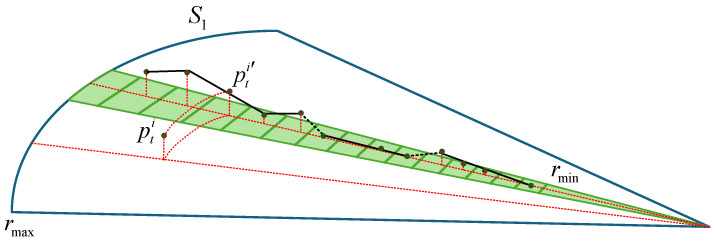
Point cloud coordinate transformation and line fitting in the first sector. The green areas represent the sub-sectors, the solid black lines show the fitted ground feature lines, and the dashed black lines indicate lines that do not meet the constraints.

Line fitting is then performed within each sector $S_{s}$. The point with the smallest $z_{t}^{i}$ in each sub-sector is selected as the initial point, and the points are sorted by their radial distance $r_{t}^{i}$. An incremental least squares method is applied to progressively fit a line z=mr+b by adding points sequentially as shown in Figure 2. The fitting process restarts with a new line once the inclusion of a new point causes the fitting error to exceed a predefined threshold.

Finally, ground points are segmented by evaluating the fitted lines. Among the multiple lines fitted in a sector, only those that satisfy the following criteria are considered as representing the ground:

- Slope: The slope of the line should not be too steep, as the ground inclination should be relatively moderate.
- Intercept: The intercept of the line should not be too large, as ground height is not expected to vary significantly.
- Fitting error and continuity: The fitting error and the continuity of adjacent lines should be within acceptable bounds, as the ground should not be excessively rough.

After identifying the ground-representing lines in each sector, every original 3D point is evaluated by computing its Euclidean distance to the nearest ground line. Points whose distance falls below a set threshold are classified as ground points and removed from subsequent feature extraction. This procedure effectively filters out ground points and yields a non-ground point cloud $P_{ng}$, thus improving the reliability of feature extraction and enhancing the overall stability and accuracy of the odometry.

### 4.2. Feature Point Extraction

Feature-based point cloud matching methods [11] significantly reduce computational load while preserving the geometric structure of the environment. The point clouds used for feature extraction are first pre-processed by de-skewing using IMU data, which enhances the accuracy of feature matching. These methods also capture global structural information between point clouds, exhibiting strong robustness against noise and partial occlusion.

To select representative features from the point cloud, the smoothness of each point is used to classify LiDAR data. The smoothness is computed as described in Equation (2):

(2) $$c=\frac{1}{\left|\Omega \right|\cdot \left\|p_{t}^{i}\right\|}\left\|\sum_{j\in N,j\neq i}\left(p_{t}^{i}-p_{t}^{j}\right)\right\|$$

where $p_{t}^{i}$ is one point in $P_{ng}$, Ω denotes the set of neighboring points around $p_{t}^{i}$ within a local window, $p_{t}^{j}$ is another point in Ω distinct from $p_{t}^{i}$, and $\left|\Omega \right|$ is the number of points in Ω. Each scan line of the multi-beam LiDAR is evenly divided into four sectors. Points within each sector are sorted by their smoothness. Points with smoothness values above a defined threshold are classified as edge features, while those below another threshold are classified as planar features. To ensure an even distribution of features across the environment, each sector contributes at most two edge points and four planar points, which are added to the edge feature set $F_{t}^{e}$ and planar feature set $F_{t}^{p}$, respectively.

Using the robot pose from the previous frame, IMU pre-integration results, and the LiDAR extrinsic parameters, the feature sets are transformed into the world coordinate frame to form a LiDAR keyframe $\left\{{\bar{F}}_{t}^{e},{\bar{F}}_{t}^{p}\right\}$. A sliding-window approach is adopted to construct a local map $M_{t}=\{M_{t}^{e},M_{t}^{p}\}$ from a fixed number of recent keyframes. The local map consists of an edge map $M_{t}^{e}={\bar{F}}_{t}^{e}\cup {\bar{F}}_{t-1}^{e}\cup \cdots \cup {\bar{F}}_{t-n}^{e}$ and a planar map $M_{t}^{p}={\bar{F}}_{t}^{p}\cup {\bar{F}}_{t-1}^{p}\cup \cdots \cup {\bar{F}}_{t-n}^{p}$, which are then used for robust and efficient scan matching in subsequent stages.

### 4.3. Pose Estimation Based on Multi-Sensor Fusion

After completing the preprocessing of IMU and LiDAR data described in the preceding sections, a multi-sensor fusion-based pose estimation scheme is constructed to achieve accurate and stable localization.

#### 4.3.1. LiDAR Feature Matching and Odometry Construction

Once a new LiDAR scan has been processed into a LiDAR feature keyframe $\left\{{\bar{F}}_{t+1}^{e},{\bar{F}}_{t+1}^{p}\right\}$ through the aforementioned steps, it is matched against the local map $M_{t}=\{M_{t}^{e},M_{t}^{p}\}$ using the ICP algorithm. For edge features, a point-to-line distance metric is employed, while for planar features, a point-to-plane distance metric is used. The corresponding residuals are formulated as follows:

(3) $$d_{e_{k}}=\frac{\left\|\left(p_{t+1,k}^{e}-p_{t,u}^{e}\right)\times \left(p_{t+1,k}^{e}-p_{t,v}^{e}\right)\right\|}{\left\|p_{t,u}^{e}-p_{t,v}^{e}\right\|}$$

(4) $$d_{p_{k}}=\frac{\left(p_{t+1,k}^{p}-p_{t,u}^{p}\right)\cdot \left[\left(p_{t,u}^{p}-p_{t,v}^{p}\right)\times \left(p_{t,u}^{p}-p_{t,w}^{p}\right)\right]}{\left\|\left(p_{t,u}^{p}-p_{t,v}^{p}\right)\times \left(p_{t,u}^{p}-p_{t,w}^{p}\right)\right\|}$$

where $p_{t,u}^{e},p_{t,v}^{e},p_{t,u}^{p},p_{t,v}^{p},p_{t,w}^{p}\in M_{t}$ denote the closest points in the local map to the new edge point $p_{t+1,k}^{e}$ or planar point $p_{t+1,k}^{p}$. The edge residual $d_{ek}$ represents the distance from the point to the local edge line, while the planar residual $d_{pk}$ represents the distance to the local planar surface.

The objective is to estimate the optimal pose transformation represented by the rotation matrix $R_{t,t+1}$ and translation vector $T_{t,t+1}$ for the current frame by solving the optimization problem:

(5) $$R_{t,t+1},T_{t,t+1}=\underset{R,T}{\arg\min}\left(\sum_{k\in {\bar{F}}_{t+1}^{e}}d_{e_{k}}^{2}+\sum_{k\in {\bar{F}}_{t+1}^{p}}d_{p_{k}}^{2}\right)$$

In this formulation, edge and planar residuals are weighted by a robust kernel that assigns higher weights to smaller residuals and down-weights potential outliers, following the practice in LIO-SAM. This nonlinear least-squares problem is solved using the Gauss-Newton method [41] to obtain the optimal $R_{t,t+1}$ and $T_{t,t+1}$. The optimization is initialized using the pose predicted by IMU pre-integration and iterates until the pose update increments fall below a predefined threshold or a maximum number of iterations is reached. Given the pose of the previous frame, the relative transformation is computed and incorporated into the optimization as a LiDAR factor.

At this stage, a LiDAR-inertial odometry system has been constructed. It takes IMU pre-integration outputs and LiDAR point clouds as inputs, performs ground point filtering, feature extraction, and point cloud matching, and outputs frame-to-frame pose estimates. However, in this formulation, IMU data are primarily used for motion distortion correction and to provide a motion prior, without being fully integrated into the optimization process. As a result, the system operates in a loosely coupled manner, leading to an over-reliance on LiDAR measurements and reduced robustness, particularly in challenging environments.

#### 4.3.2. Tightly Coupled Multi-Sensor Fusion via Factor Graph Optimization

**Figure 3:**
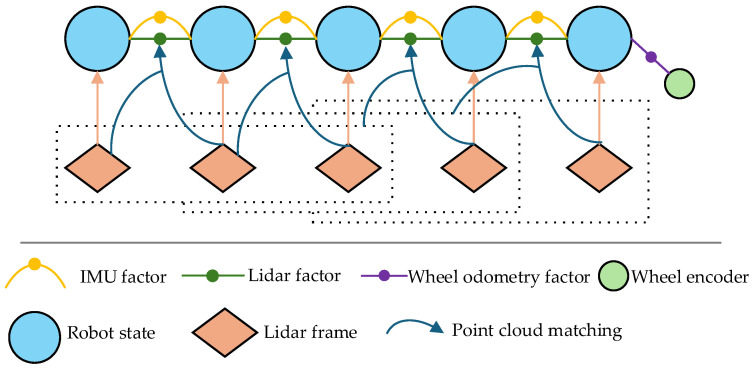
Factor graph-based optimization framework.

To overcome this limitation, we adopt a factor graph–based optimization framework, as illustrated in Figure 3, to tightly integrate measurements from multiple sensors in a unified probabilistic formulation. The factor graph incorporates three types of factors: IMU factors derived from pre-integrated IMU measurements, wheel odometry factors constructed from wheel speed measurements, and LiDAR factors obtained from scan-matching residuals.

The multi-sensor fusion and state estimation problem is formulated as a large-scale nonlinear least-squares optimization, which jointly minimizes the residuals associated with all sensor measurements and robot states. IMU and LiDAR factors directly constrain the robot’s pose transformation, while the wheel odometry factor—providing velocity observations—imposes constraints on the robot’s velocity estimate, thereby enhancing motion consistency and stability.

Regarding the uncertainty modeling, the covariance matrices for the IMU and LiDAR factors follow the standard practice in LIO-SAM, with noise parameters derived from sensor specifications and empirical tuning. The wheel odometry factor covariance is adapted online according to the road surface condition, i.e., low on smooth roads and high on rough or slippery surfaces, reflecting the expected measurement confidence. To bound the computational cost, a pseudo-sliding window strategy is adopted: the factor graph is reset every 100 keyframes, which implicitly marginalizes old states and keeps the graph size manageable for real-time operation. Loop closure factors are intentionally not included in our framework, as large-scale road environments often contain long traversals without repeated visits to previously mapped areas, making loop closure detection unreliable and impractical in such scenarios.

The resulting factor graph is optimized incrementally using the iSAM2 algorithm [42], enabling efficient real-time inference. This process yields a high-accuracy and high-robustness estimate of the robot’s pose, completing the construction of a tightly coupled multi-sensor fusion odometry suitable for robust localization in large-scale environments.

## 5. Drift Correction Using HMM and Topological Map

As analyzed in Section 2, a critical limitation of existing localization methods is the accumulation of drift over time. In this section, we present a drift correction scheme based on digital map matching. The proposed method leverages open-source digital maps together with the robot trajectory estimated by the multi-sensor fusion odometry to reduce or even eliminate the impact of accumulated drift in large-scale robot localization.

### 5.1. Overall Framework

**Figure 4:**
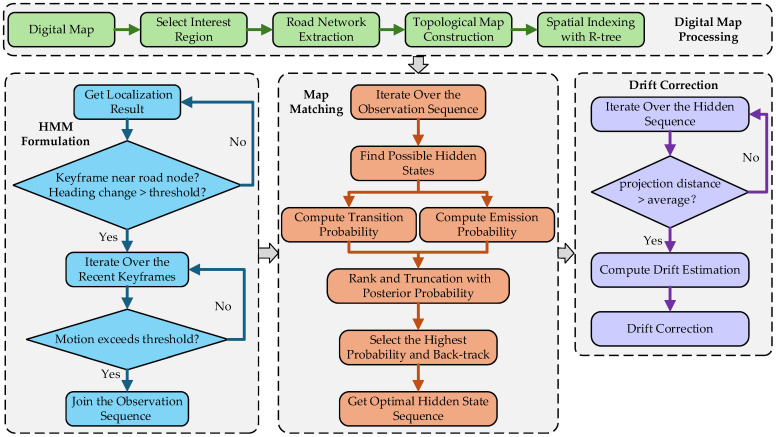
The overall pipeline of drift correction using HMM and topological map matching.

The overall pipeline of the proposed drift correction method is illustrated in Figure 4. First, an open-source digital map (e.g., OpenStreetMap) is acquired and preprocessed to extract road information, which is then converted into a topological road network to facilitate mathematical representation and reasoning. A spatial R-tree index is then constructed for each road segment in the topological map, enabling efficient spatial queries and rapid retrieval of candidate segments during map matching.

When predefined triggering conditions are satisfied, recent LiDAR keyframes are matched against nearby road segments in the topological map to identify the road segment on which the robot is currently traveling. The absolute positional constraints provided by the digital map are then used to adjust the robot’s pose estimate, thereby correcting accumulated drift.

### 5.2. Topological Map Construction

To enable drift correction using digital maps, preprocessing is required to facilitate subsequent operations. This section describes the conversion of open-source digital maps into a topological representation, which consists of three main steps: road information extraction, topological road network construction, and spatial indexing using an R-tree.

First, road information is extracted from the digital map. Digital maps typically contain diverse geographic elements, such as buildings, administrative boundaries, and road networks. In this work, the OSMNX library is used to parse OpenStreetMap (OSM) data. A region of interest is selected on the OSM website, and the corresponding map file is exported in XML format. The file contains a series of geographic tags, where each node element represents a landmark defined by its longitude (lon) and latitude (lat) coordinates. Since OSM includes heterogeneous and complex geographic information, much of which is irrelevant for road-based navigation, further filtering is performed to retain only road-related data. The way element in OSM contains a unique ID, a list of node IDs that make up the way, and a set of descriptive tags. The highway key within these tags indicates whether a way corresponds to a road. Using this key–value pair, the road network can be efficiently extracted from the raw OSM dataset.

After extracting road data, the map is converted into a topological representation. Following graph-theoretic principles, road intersections are modeled as nodes N, while road segments connecting them are modeled as edges M. The weight of each edge is defined as its geometric length. Consequently, the digital map is represented as a weighted graph G=(N,M).

**Figure 5:**
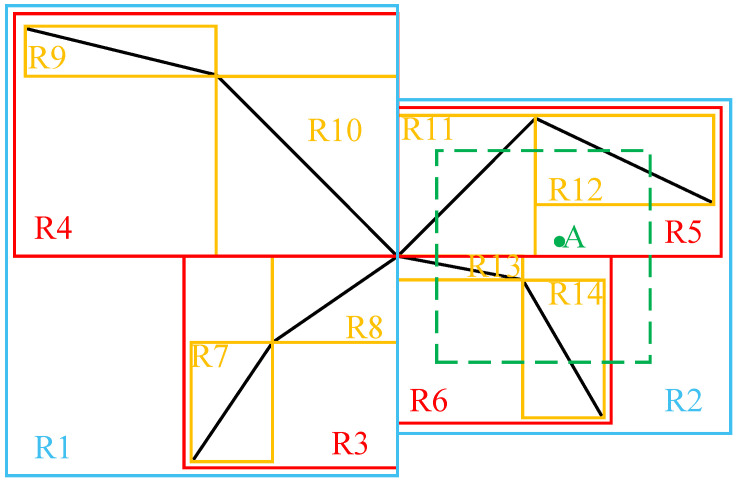
Topological map spatial index with R-tree. The black polyline represents the topological road network, multi-colored boxes indicate hierarchical MBRs at different levels, the green point A denotes the query location, and the green dashed box marks the candidate region.

To enable efficient spatial queries during trajectory matching, the road segments are indexed using an R-tree. The R-tree organizes spatial objects hierarchically using Minimum Bounding Rectangles (MBRs). Leaf nodes correspond to individual road segments, while higher-level nodes recursively group spatially proximate MBRs, thereby partitioning the entire map space as shown in Figure 5. When querying nearby road segments, a candidate bounding box centered at the query location is generated based on a predefined search radius. The R-tree is traversed from the root to the leaf nodes, retrieving only those road segments whose MBRs intersect the query box. By grouping spatially adjacent segments under common parent nodes, the R-tree significantly reduces search complexity and improves matching efficiency.

### 5.3. Drift Correction

After preprocessing, the open-source OSM data are converted into a topological road network and indexed using an R-tree. This subsection presents a cumulative drift correction method based on matching the robot’s estimated trajectory to the topological map. By filtering LiDAR keyframes to construct a representative trajectory and aligning it with the digital map, the system estimates accumulated drift and maintains low localization error over long-term operation.

#### 5.3.1. Formulation as a Hidden Markov Model

The problem of matching selected LiDAR keyframe positions to the topological road network is formulated as a decoding problem in an HMM. The sequence of filtered LiDAR keyframe poses constitutes the observation sequence $O=(O_{1},O_{2},\cdots ,O_{n})$ while the corresponding true positions on the road network form the hidden state sequence $S=(S_{1},S_{2},\cdots ,S_{n})$.

Intuitively, the closer an observation lies to a road segment, and the more consistent the inter-frame motion is with the corresponding displacement along the road network, the higher the probability that the observation corresponds to that segment. Accordingly, the emission probability is modeled as a function of the distance between the observation and its projection onto the road network, while the transition probability is derived from the discrepancy between consecutive observations and the distance traveled along the road network. The HMM satisfies the standard assumptions: Markov property is the current hidden state depends only on the previous hidden state; observation independence is each observation depends only on the current hidden state.

LiDAR keyframes are selected according to the following criteria: consecutive keyframes are generated when the translational displacement exceeds 0.3 m or the rotational change exceeds 10°. A feature trajectory starts and ends when the keyframe lies within 5 m of a road network node and the heading change between the start and end exceeds 30°. Only sequences satisfying these conditions are used as observation sequences. This strategy ensures that map matching is performed using informative and well-distributed keyframes, thereby improving both efficiency and robustness.

#### 5.3.2. Topological Map Matching

During map matching, each observation corresponds to multiple candidate hidden states. To identify the most probable hidden state sequence given the observation sequence, the decoding problem is solved using the Viterbi algorithm.

Let the current observation sequence be $O=(O_{1},O_{2},\cdots ,O_{n})$ where $O_{n}$ corresponds to the most recent keyframe. Starting from the first observation $O_{1}$, the R-tree index is queried to retrieve all road segments within a predefined search radius. Each candidate segment is projected onto the observation to generate a set of possible hidden states $S_{1,j}$. The emission probability for each candidate is computed using a Gaussian model:

(6) $$p(O_{i}|S_{i,j})=e^{-\frac{{\left\|O_{i}-S_{i,j}\right\|}^{2}}{2\sigma_{O}^{2}}}$$

where $\sigma_{O}$ is the standard deviation of the projection error. Candidates are ranked by probability, and only the most likely states are retained via truncation.

**Figure 6:**
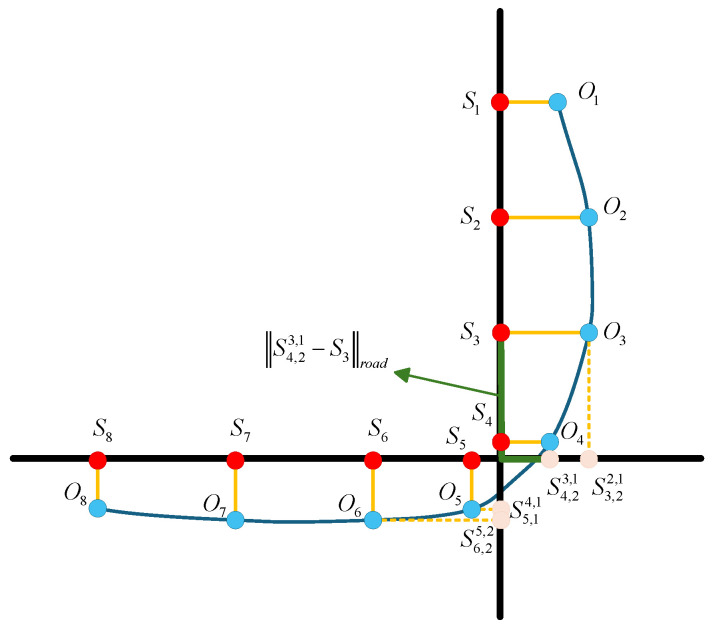
Topological map matching results. The black line represents the road network, the blue line denotes the estimated trajectory, blue points indicate observations, red points are optimal hidden states, and light red points represent candidate hidden states.

Since the hidden state at the next time step can only lie on the same road segment as the previous state or on a directly connected segment, the transition search space is further constrained. For observation $O_{2}$, the transition probability from $S_{1,j}$ to $S_{2,k}^{1,j}$ is defined as:

(7) $$p(S_{i,j}|S_{i+1,k}^{i,j})=e^{-\frac{{\left(\left\|O_{i+1}-O_{i}\right\|-{\left\|S_{i,j}-S_{i+1,k}^{i,j}\right\|}_{road}\right)}^{2}}{2\sigma_{S}^{2}}}$$

where ${\left\|S_{i,j}-S_{i+1,k}^{i,j}\right\|}_{road}$ is the distance along the road network like green line in Figure 6 (including junction paths if the segments differ), if $S_{i,j}$ and $S_{i+1,k}^{i,j}$ lie on different segments, the distance includes the path through the connecting junction. In addition, $\sigma_{S}$ is the standard deviation of the error that between consecutive observations and the ${\left\|S_{i,j}-S_{i+1,k}^{i,j}\right\|}_{road}$.

The posterior probability of each candidate hidden state is updated recursively using $p(S_{i,k})=p(S_{i-1,j})p(S_{i-1,j}|S_{i,k}^{i-1,j})p(O_{i}|S_{i,k})$, followed by ranking and truncation. After processing all frames, the hidden state with the highest probability is selected, and back tracking yields the optimal hidden state sequence $S=(S_{1},S_{2},\cdots ,S_{n})$, completing the map trajectory alignment.

#### 5.3.3. Drift Correction Implementation

Once the k-th map matching yields the new matched road positions $S=(S_{1},S_{2},\cdots ,S_{n})$ corresponding to the observed trajectory $O=(O_{1},O_{2},\cdots ,O_{n})$. Drift is estimated using only those frames whose projection distance exceeds the average projection distance $\mu =\sum_{i=1}^{n}\left\|O_{i}-S_{i}\right\|/n$. Since the robot’s true trajectory cannot perfectly align with the road network, this approach mitigates the resulting mismatch.

Let the k map matching drift $\Delta_{k}$ be updated incrementally as:

(8) $$a_{i}=\left\{\begin{array}{cc} 0 & \left\|O_{i}-S_{i}\right\|<\mu \\ 1 & otherwise \end{array}\right.$$

(9) $$\Delta_{k}=\Delta_{k-1}+\frac{1}{\lambda }\sum_{i=1}^{n}a_{i}(O_{i}-S_{i})$$

where λ is the number of observations $O_{i}$ whose projection distance exceeds μ. Since the road network provides only latitude and longitude information, the correction is inherently limited to the horizontal plane. In ground robot road navigation scenarios, vertical deviations are typically small and have less impact on overall performance, and planar accuracy remains the primary concern, this makes horizontal-only correction $\Delta_{k}=(\Delta x_{k},\Delta y_{k})$ a reasonable design choice. Finally, the estimated drift $\Delta_{k}$ is subtracted from the current localization output to obtain the corrected pose, effectively compensating for accumulated drift.

## 6. Experiment Results

To validate the effectiveness of the proposed multi-sensor fusion odometry and drift correction method, a series of experiments were conducted and analyzed. The experimental site and hardware setup are described in Section 3. To evaluate performance in large-scale and complex road environments, two datasets were collected in different campus settings: the College Dataset and the School Dataset.

**Figure 7:**
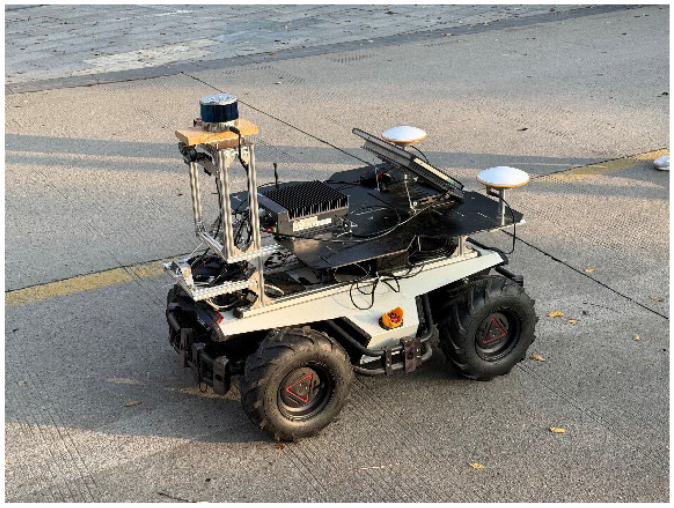
Self-built unmanned vehicle platform.

**Figure 8:**
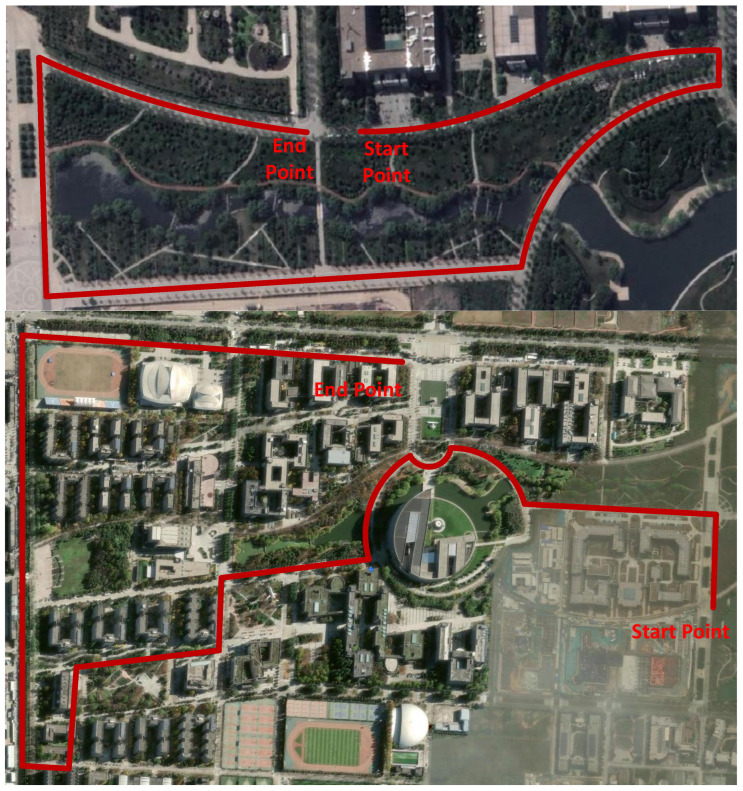
Satellite images of the actual scenes for both datasets with the College Dataset at the top and the School Dataset at the bottom. The red line is UGV’s trajectory.

The sensor platform was a self-built unmanned ground vehicle (UGV), as shown in Figure 7. Both datasets were recorded on various road types within the campuses, including structured main roads and unstructured branch roads. Figure 8 shows satellite imagery of the two test areas with the UGV trajectories overlaid in red. To highlight the role of road network matching in drift correction, the UGV was driven along routes closely aligned with the OpenStreetMap (OSM) road network during data collection. Unless otherwise specified, the following default covariance settings were used throughout all experiments. For the LiDAR and IMU factors, the covariance matrices followed the standard configuration in LIO-SAM. For the newly introduced wheel odometry factor, the pose covariance was set to $\mathrm{diag}[0.1,0.1,1\times 10^{6},1\times 10^{6},1\times 10^{6},0.3]$, and a velocity covariance was set to $\mathrm{diag}[0.1^{2},0.1^{2},1\times 10^{6}]$.

To evaluate the individual contributions of the three proposed modules, ground point filtering, the wheel odometry factor, and topological map matching, an ablation study was first conducted on the College Dataset. To assess the generalization capability of the localization method, experiments were then performed on the public KITTI odometry benchmark (Sequence 05), where the proposed algorithm was compared with LeGO-LOAM, LIO-SAM, and FAST-LIO2. Subsequently, the topological map matching module, as the core component for drift correction, was analyzed in depth across all datasets to examine its correction behavior. Finally, to demonstrate the advantage of the proposed method in large-scale road environments, a long-distance experiment was conducted on the School Dataset, comparing the proposed method against LeGO-LOAM, LIO-SAM and FAST-LIO2.

### 6.1. Ablation Study

To evaluate the individual contributions of the three proposed modules—ground point filtering, wheel odometry factor, and topological map matching—an ablation study was conducted on the College Dataset. Four configurations were compared:

- LIO: the baseline LiDAR-inertial odometry without any of the proposed modules.
- LIO + Wheel: LIO augmented with the wheel odometry factor.
- LIO + Wheel + Ground Filter: both wheel odometry and Line-Fit ground filtering.
- Full Method (LIO + Wheel + Ground Filter + Map Matching): the complete proposed system.

All experiments were performed on the same College Dataset. To quantitatively evaluate localization accuracy, the estimated trajectories were compared with the ground-truth trajectory provided by the CGI410 high-precision integrated navigation system. Both the ground-truth and estimated trajectories were recorded at 10 Hz and aligned to the same global coordinate frame using the initial GNSS pose obtained during system initialization. Localization accuracy was assessed using the Absolute Trajectory Error (ATE) [43] metrics, including the maximum error (Max), mean absolute error (MAE), root mean square error (RMSE), and standard deviation (STD) of the Euclidean distance between the estimated and ground-truth positions. Let $p_{i}^{est}$ and $p_{i}^{gt}$ denote the estimated and ground-truth positions at time step i on the horizontal plane. N denotes the total number of samples. The MAE, RMSE, and STD are defined as follows:

(10) $$\mathrm{MAE}=\frac{1}{N}\sum_{i=1}^{N}\left\|p_{i}^{est}-p_{i}^{gt}\right\|$$

(11) $$\mathrm{RMSE}=\sqrt{\frac{1}{N}\sum_{i=1}^{N}{\left\|p_{i}^{est}-p_{i}^{gt}\right\|}^{2}}$$

(12) $$\mathrm{STD}=\sqrt{\frac{1}{N-1}\sum_{i=1}^{N}{\left(\left\|p_{i}^{est}-p_{i}^{gt}\right\|-\mathrm{MAE}\right)}^{2}}$$

**Figure 9:**
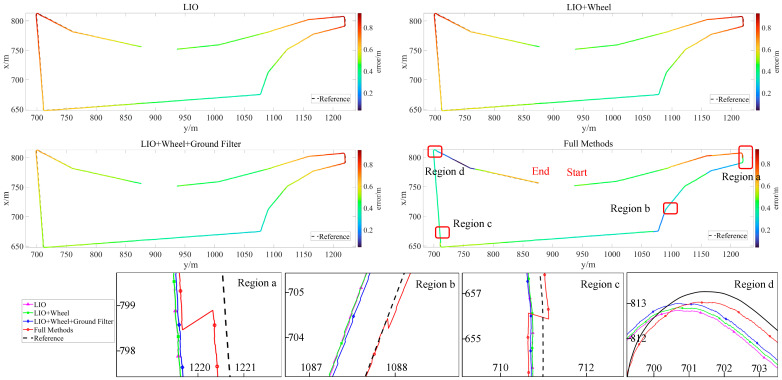
Ablation study trajectories on the College Dataset. The upper panel displays the global trajectories estimated by LIO, LIO + Wheel, LIO + Wheel + Ground Filter, and the Full Method, together with the ground-truth reference. Four representative regions (a–d) are enlarged in the lower panels to illustrate the progressive reduction in drift as each module is activated.

Figure 9 shows the global localization trajectories estimated by the four configurations on the College Dataset, along with enlarged views of four key regions (labeled a–d). These regions highlight the effectiveness of topological map matching and the influence of each module after the vehicle has traveled a certain distance. In the figure, the magenta line with triangle markers represents LIO; the green line with square markers represents LIO + Wheel; the blue line with diamond markers represents LIO + Wheel + Ground Filter; and the red line with circle markers represents the Full Method. The black line denotes the ground-truth trajectory.

The zoomed-in views in Figure 9. a–d reveal how the localization error evolves and how each module contributes to drift correction. In region (a), (b) and (c), immediately after the vehicle passes the first sharp turn, the Full Method leverages topological map matching to directly correct the drifted estimate back to the vicinity of the reference trajectory, whereas the other variants remain visibly deviated. As the travel distance increases, cumulative drift becomes more pronounced for the configurations without map matching; nevertheless, the Full Method continues to suppress this drift through repeated topological corrections. Finally, after the vehicle has traversed the long-range route, region (d) clearly demonstrates the progressive benefit of each module: LIO + Wheel, by incorporating the wheel odometry factor to constrain the robot velocity, achieves a smaller error than the baseline LIO; LIO + Wheel + Ground Filter further removes ground points and reduces environmental noise, thereby improving scan-matching accuracy and yielding an even lower error; and the Full Method, which continuously corrects the accumulated drift via topological map matching, attains a substantial improvement in localization accuracy and remains the closest to the reference trajectory among all configurations.

To quantify the contribution of each module, the average Absolute Trajectory Error (ATE) statistics over the seven repeated runs are summarized in Table 1, and the detailed data for each run are shown in Appendix A. Among the three proposed modules, topological map matching yields the most significant improvement: it reduces the mean absolute error (MAE) by 17.4% compared with the configuration without map matching (LIO + Wheel + Ground Filter), and also substantially improves the other error metrics. The only exception is the STD of the error, which is slightly higher for the Full Method. This is an expected consequence of the discrete jump corrections performed by map matching—the estimate is occasionally pulled directly to a much more accurate position, resulting in a mixture of very small and relatively large errors and therefore a larger dispersion. The ground point filtering module consistently improves all ATE metrics. By removing ground points, it reduces environmental noise and enhances the accuracy of scan matching, which is the core of the LiDAR-inertial odometry, thus leading to an overall improvement in localization performance. The wheel odometry factor contributes a moderate yet consistent improvement. By incorporating velocity observations, it constrains the robot’s motion and suppresses short-term drift, particularly during aggressive turns or in feature-sparse segments, thereby complementing the other modules and enhancing overall robustness. The statistical significance of the improvements was further assessed using the Wilcoxon signed-rank test. Both the ground point filtering and the topological map matching modules demonstrated strong significance, with a *p*-value of 0.0156. The wheel odometry factor also exhibited statistical significance, although at a weaker level, with a *p*-value of 0.0313.

In summary, the ablation study confirms that all three proposed modules contribute positively to localization accuracy. Topological map matching provides the largest improvement by directly correcting accumulated drift, while ground point filtering enhances scan-matching quality and yields a consistent overall gain. The wheel odometry factor offers a moderate but stable improvement by constraining short-term motion drift, and its combination with the other two modules achieves the best performance across all metrics except for STD.

### 6.2. Evaluation on Public Benchmark

To assess the generalization capability of the proposed method, experiments were conducted on a public benchmark dataset. Although most public datasets do not include wheel odometry data, the ablation study in Section 6.1 has demonstrated that the wheel odometry factor provides a moderate but non-essential improvement to the overall accuracy; its absence does not fundamentally compromise the localization performance. Testing on public benchmarks without wheel odometry can therefore still meaningfully reflect the effectiveness of the proposed approach. The KITTI odometry Sequence 05 was selected for this evaluation, as it covers a total length of 2205 m in an urban road environment, closely matching the large-scale road environments targeted in this work. The proposed method was compared with three mainstream baselines: LeGO-LOAM, LIO-SAM, and FAST-LIO2. For all algorithms, GNSS was used exclusively during initialization to align the coordinate frame, and loop closure detection was disabled, to better emulate the long-range, large-scale localization conditions without global corrections.

**Figure 10:**
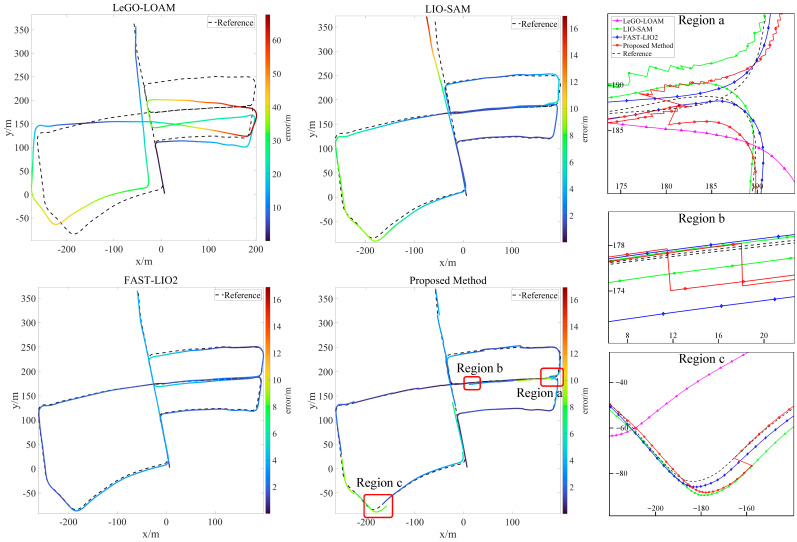
Evaluation on the KITTI Sequence 05 public benchmark. Left: error heatmaps of the global trajectories estimated by LeGO-LOAM, LIO-SAM, FAST-LIO2, and the proposed method, with warmer colors indicating larger deviations from the reference. Right: enlarged views of three representative regions a–c. In the trajectory plots, the magenta line with triangle markers denotes LeGO-LOAM, the green line with square markers denotes LIO-SAM, the blue line with diamond markers denotes FAST-LIO2, and the red line with circle markers denotes the proposed method.

Figure 10 (left) presents the error heatmaps of the four compared algorithms. It is evident that both LeGO-LOAM and LIO-SAM accumulate substantial drift as the travel distance increases, while FAST-LIO2 and the proposed method maintain the error at a considerably lower level. This performance advantage stems from two factors: first, the ground filtering module enhances the accuracy of the underlying LiDAR-inertial odometry, thereby reducing the baseline drift rate; second, the topological map matching module directly corrects the accumulated drift, effectively suppressing the growth of error over long trajectories. The effectiveness of these corrections is clearly illustrated in the enlarged regions (b) and (c), where the proposed trajectory stays tightly aligned with the reference.

However, region (a) reveals a limitation related to the quality of the OSM road network. Since the KITTI Sequence 05 was not originally recorded with OSM alignment in mind, the reference trajectory does not closely follow the OSM road centerlines. In this specific turn, the vehicle approaches from below and turns left; after the turn, its path deviates upward from the OSM road geometry. Consequently, the map-matching correction pulls the estimate toward the OSM road network, which—being offset from the actual driven path—introduces a noticeable residual error. In contrast, the trajectory approaching from above and turning right follows the OSM road network more closely, and the correction there yields a much smaller error. This observation indicates that the proposed method relies on a reasonable level of OSM map accuracy and alignment with the actual driving path. Nevertheless, in the vast majority of the trajectory, the map-matching correction remains reliable and significantly improves overall localization accuracy.

Table 2 summarizes the ATE statistics for all four methods. LeGO-LOAM exhibits the largest drift by a considerable margin, with an average error of 27.603 m and a maximum error exceeding 67 m, confirming that even with ground point filtering, a LiDAR-only odometry cannot suppress the accumulated drift over long distances. LIO-SAM significantly reduces the average error to 3.812 m, but its maximum error remains above 16 m, indicating that drift continues to accumulate over the trajectory. FAST-LIO2 achieves the lowest error among the baselines (average 2.586 m, maximum 5.763 m), benefiting from its robust LiDAR-inertial odometry. The proposed method yields an average error of 2.779 m, which is very close to that of FAST-LIO2 despite operating without wheel odometry on this dataset, and its RMSE (3.642 m) remains competitive. The slightly larger maximum error (10.023 m) and variance can be attributed to the occasional mis-correction caused by OSM misalignment, as discussed for region (a) in Figure 10. Overall, these results demonstrate that the proposed approach generalizes well to a public benchmark and remains highly competitive with state-of-the-art methods.

In summary, the evaluation on the KITTI Sequence 05 public benchmark demonstrates that the proposed method generalizes well beyond the proprietary campus datasets. Even in the absence of wheel odometry data, it maintains localization accuracy competitive with FAST-LIO2 and substantially outperforms LeGO-LOAM and LIO-SAM. However, the occasional mis-correction observed in region (a) of Figure 10 highlights a sensitivity to the alignment between the OSM road network and the actual driven path. When the vehicle trajectory deviates from the mapped road centerline, the map-matching correction may pull the estimate toward the map and temporarily increase the error. This motivates a deeper investigation into the correction behavior of the topological map matching module, which is presented in the following section.

### 6.3. Analysis of Topological Map Matching Correction

**Figure 11:**
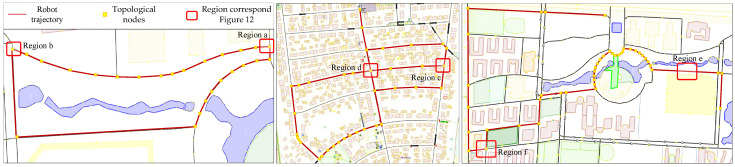
OSM maps of all datasets: College Dataset (**left**), KITTI05 (**middle**) and School Dataset (**right**). Red lines show the UGV trajectories as matched onto the OSM map.; yellow squares denote topological nodes. Regions a–f correspond to Figure 12. (a)–(f).

This section evaluates the proposed topology-based map matching drift correction method on all datasets. Figure 11 shows the OSM and road networks of the three test areas, with the UGV trajectories plotted in red and topological nodes marked as yellow squares.

**Figure 12:**
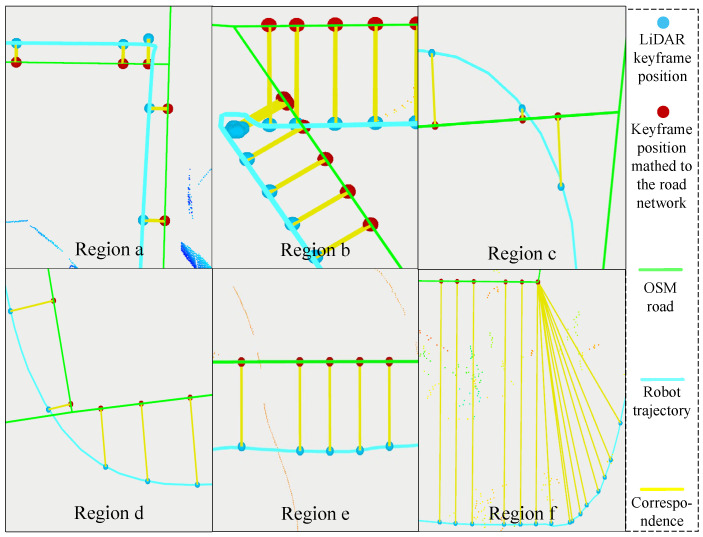
Local enlarged views of map matching, (a)–(f) corresponding to regions a–f in Figure 11. Green lines denote OSM road segments, blue dots are LiDAR keyframe positions, red dots indicate keyframe positions successfully matched to the road network, and the cyan curve is the robot trajectory.

Figure 12 illustrates the topological map matching results corresponding to the six regions (a–f) highlighted in Figure 11. In the figure, green lines denote the OSM road segments, blue dots represent the LiDAR keyframe positions, red dots indicate keyframes that are successfully matched to the OSM road network, and the cyan curve shows the keyframe trajectory. In all regions except (c), the algorithm reliably matches keyframes to the OSM network on both straight and curved road sections, even when a certain lateral deviation exists between the trajectory and the map. However, in region (c), the vehicle path deviates substantially from the OSM road geometry due to inaccuracies in the OSM map, causing the matching to fail. This observation reinforces the conclusion drawn from the KITTI experiment: the topological map matching module is generally robust, but its performance is contingent on a reasonable alignment between the driven path and the OSM road network.

To quantitatively assess matching performance, we analyzed the localization error before and after each successful match. Specifically, the difference between the error at the matched keyframe and that at the immediately preceding moment was computed, representing the single-match correction magnitude $\left|\Delta_{k}\right|$. The mean correction is $\left|{\bar{\Delta }}_{k}\right|$. The correction ratio relative to the pre-match error was also calculated as $\left|{\bar{\Delta }}_{k}\right|/e_{k}$ where $e_{k}$ is the error between the keyframe and reference. Ten matches were performed on the College Dataset, twenty-seven on KITTI 05, and forty-one on the School Dataset. The quantitative results are summarized in Table 3.

As shown in Table 3, the number of successful map matching corrections increases with travel distance, particularly as the vehicle undergoes more large-angle turns. On the College and School datasets, where the OSM maps were manually corrected for higher accuracy, the correction ratio remains at approximately 50%. This means that, on average, each topological map matching operation eliminates roughly half of the accumulated drift. Furthermore, the mean correction magnitude grows with the travel distance, reflecting the progressive accumulation of odometry drift. In contrast, on the KITTI 05 dataset, the OSM map was directly downloaded without manual refinement, resulting in lower map quality and a reduced correction ratio of 12.3%. Despite this lower ratio, the map matching still contributes meaningfully to drift reduction: when the accumulated error reaches the meter level after long-distance travel, even a 12% correction can translate to a decimeter-level improvement in localization accuracy.

### 6.4. Large-Scale Localization on School Dataset

To highlight the advantage of the proposed method in large-scale road environments, a long-distance experiment was conducted on the School Dataset, which spans 3645 m without any loop closure. GNSS data was used exclusively during initialization for coordinate frame alignment, simulating the typical large-scale road scenario where GNSS is frequently unavailable due to tall buildings, dense tree canopies, or similar obstructions.

**Figure 13:**
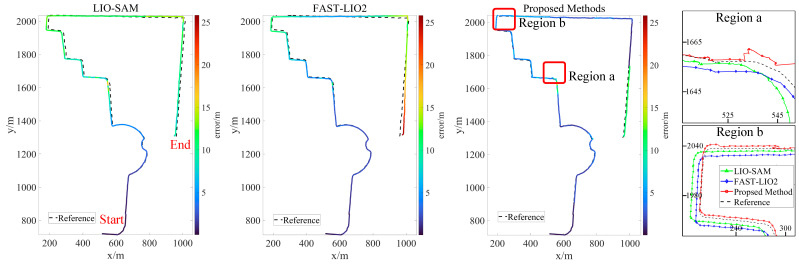
Long-scale localization results on the School Dataset. (**Left**): error heatmaps of the trajectories estimated by LIO-SAM, FAST-LIO2, and the proposed method, with warmer colors indicating larger deviation from the reference. (**Right**): magnified views of regions (a) and (b). In the trajectory plots, the green line with triangle markers denotes LIO-SAM, the blue line with diamond markers denotes FAST-LIO2, the red line with circle markers denotes the proposed method, and the black line represents the ground-truth trajectory.

The proposed method was compared against LIO-SAM and FAST-LIO2 under identical conditions. Figure 13 presents the error heatmaps of the global trajectories (left) and enlarged views of two representative regions (a and b, right).

As shown in the error heatmaps, both LIO-SAM and FAST-LIO2 suffer from pronounced cumulative drift. In particular, FAST-LIO2 exhibits a steadily increasing error that exceeds 25 m over the long trajectory. In contrast, the proposed method maintains a much lower error level throughout the entire route. This superior performance is attributed to three synergistic factors: first, the ground point filtering module improves the accuracy of the LiDAR odometry, reducing the per-frame drift; second, the wheel odometry factor provides additional motion constraints that further slow down error accumulation; and third, the topological map matching module continuously corrects the accumulated drift, preventing it from growing to large magnitudes.

The effectiveness of the map-matching correction is clearly visible in the magnified regions. In region (a), after the vehicle executes a sharp turn, the map-matching correction pulls the drifted trajectory back to the vicinity of the reference. As the travel distance increases further, region (b) shows that both LIO-SAM and FAST-LIO2 have already accumulated errors on the order of 10 m, whereas the proposed method keeps the error at or below 5 m through repeated topological corrections.

Table 4 summarizes the ATE statistics for the three methods on the School Dataset. The proposed method achieves an average error of 4.199 m, representing a reduction of 48.1% compared to LIO-SAM (8.086 m) and 44.2% compared to FAST-LIO2 (7.531 m). The RMSE follows a similar trend, with the proposed method attaining 5.343 m versus 9.188 m (LIO-SAM) and 9.621 m (FAST-LIO2). Notably, FAST-LIO2 yields the largest maximum error (25.837 m) and STD (35.859 m^2^) among all methods, indicating that while its average performance is competitive, its drift grows more aggressively in certain segments of this long-distance, loop-free trajectory. This behavior aligns with the error heatmap in Figure 13, where FAST-LIO2 exhibits steadily increasing warm-colored regions as the travel distance grows. In contrast, the proposed method maintains the lowest maximum error (16.271 m) and the smallest variance, confirming that the combination of ground filtering, wheel odometry, and topological map matching not only reduces the overall drift but also improves stability throughout the entire 3645 m traversal.

In summary, the School Dataset experiment confirms that the proposed method effectively handles long-range, loop-free scenarios under GNSS-denied conditions. By synergistically combining ground point filtering, wheel odometry, and topological map matching, it reduces the average error by 48.1% relative to LIO-SAM and 44.2% relative to FAST-LIO2, while also achieving the lowest maximum error and variance. These results demonstrate strong drift resilience and robust localization in large-scale road environments.

## 7. Conclusions and Discussion

This study presents a large-scale drift-resilient localization method via multi-sensor fusion and topological map matching. To eliminate the need for constructing and maintaining high-precision prior maps, the proposed framework leverages the topological road network extracted from open-source digital maps (OSM) as the only prior information. A LiDAR-inertial odometry augmented with improved ground point filtering and a wheel odometry factor is first developed to enhance the baseline localization accuracy. A topological map matching module then corrects the accumulated odometry drift by matching the robot’s trajectory to the OSM road network. Comprehensive experiments on two proprietary campus datasets and the public KITTI 05 benchmark demonstrate the effectiveness of the proposed method. The ablation study confirms that each of the three modules—ground point filtering, wheel odometry factor, and topological map matching—contributes positively, with map matching providing the largest gain (MAE reduction of 17.4%). On the School Dataset (3645 m, loop-free), the proposed method reduces the average error by 48.1% and 44.2% compared with LIO-SAM and FAST-LIO2, respectively.

Despite these promising results, several limitations should be acknowledged. First, the localization accuracy is fundamentally dependent on the availability and quality of OSM data. The ablation study reveals that while ground point filtering and the wheel odometry factor moderately improve accuracy, the dominant performance gain comes from topological map matching. In regions without OSM coverage or where the OSM road geometry deviates significantly from the actual driven path, the drift correction may fail or even introduce additional errors, as observed on the KITTI 05 dataset. Under such conditions, the method remains functional as a LiDAR-inertial odometry, but it does not surpass existing methods. The road structure also influences performance: LiDAR-inertial odometry inherently benefits from structured road scenes with distinct geometric features, but the decisive factor determining overall localization accuracy remains OSM data quality. Second, the method incurs a non-negligible computational cost. The factor graph optimization, which constitutes the primary computational burden, requires adequate processing power. On a computer equipped with an Intel i7-10750H CPU, the system runs in real time, but its applicability to more resource-constrained platforms has not been verified. Finally, the drift correction primarily operates on planar position errors. In typical ground robot road environments, vertical deviations are relatively small compared with horizontal errors and change only modestly; nevertheless, the proposed method lacks an explicit mechanism for correcting errors in the vertical direction. Furthermore, although the wheel odometry factor introduces velocity constraints, the topological map matching module corrects only the position estimate without directly constraining velocity or orientation, which may limit performance in highly dynamic maneuvers.

Future work will focus on reducing the reliance on map accuracy, for instance, by developing robust matching strategies that tolerate significant deviations between the trajectory and the OSM network, or by integrating probabilistic map models that encode OSM accuracy. Additionally, extending the framework to support multi-platform deployment and conducting long-term evaluations under varying environmental conditions are planned.

## Figures and Tables

**Table 1:** Global localization Absolute Trajectory Error (ATE) statistics (mean of seven runs) and *p*-value of the Wilcoxon signed-rank test on the College Dataset.

Four Configurations	MAE (m)	Max Error (m)	RMSE (m)	STD (m)	Wilcoxon *p*-Value ^1^
LIO	0.5954	0.9513	0.6146	0.0232	x
LIO + Wheel	0.5795	0.9189	0.5964	0.0199	0.0313
LIO + Wheel + Ground Filter	0.5200	0.8894	0.5381	0.0192	0.0156
Full Method	0.4294	0.8103	0.4567	0.0241	0.0156

^1^ The *p*-values from the Wilcoxon signed-rank test were computed using the MAE values from the seven runs. Specifically, the *p*-value for LIO + Wheel was obtained by pairing LIO + Wheel with LIO; the *p*-value for LIO + Wheel + Ground Filter was obtained by pairing LIO + Wheel + Ground Filter with LIO + Wheel; and the *p*-value for the Full Method was obtained by pairing the Full Method with LIO + Wheel + Ground Filter.

**Table 2:** Global localization Absolute Trajectory Error (ATE) statistics on the KITTI Sequence 05.

Methods	MAE (m)	Max Error (m)	RMSE (m)	STD (m)
LeGO-LOAM	27.603	67.561	32.619	302.178
LIO-SAM	3.812	16.916	4.837	8.866
FAST-LIO2	2.586	5.763	2.848	1.422
Proposed Method	2.779	10.023	3.642	5.536

**Table 3:** Topological map matching quantification.

Dataset	Number of Matches	Mean Correction (m)	Correction Ratio
College Dataset	10	0.167	57.1%
KITTI 05	27	0.112	12.3%
School Dataset	41	0.346	46.4%

**Table 4:** Global localization Absolute Trajectory Error (ATE) statistics on the School Dataset.

Methods	MAE (m)	Max Error (m)	RMSE (m)	STD (m)
LIO-SAM	8.086	18.358	9.188	19.047
FAST-LIO2	7.531	25.837	9.621	35.859
Proposed Method	4.199	16.271	5.343	10.912

## Data Availability

The original contributions presented in this study are included in the article. Further inquiries can be directed to the corresponding author.

## Abbreviations

The following abbreviations are used in this manuscript:

GNSS	Global navigation satellite system
LiDAR	Light detection and ranging
OSM	OpenStreetMap
LIO-SAM	LiDAR inertial odometry via smoothing and mapping
LeGO-LOAM	Lightweight and ground-optimized LiDAR odometry and mapping
MAE	Mean absolute error
RMSE	Root mean square error

## Appendix A. Ablation Study—Data from Seven Runs

This appendix provides the complete Absolute Trajectory Error (ATE) statistics for all seven repeated runs of the ablation study conducted on the College Dataset. Four configurations were evaluated: LIO, LIO + Wheel, LIO + Wheel + Ground Filter, and the Full Method (LIO + Wheel + Ground Filter + Topological Map Matching).

**Table A1:** Global localization Absolute Trajectory Error (ATE) statistics of Run1 on the College Dataset.

Four Configurations	MAE (m)	Max Error (m)	RMSE (m)	STD (m)
LIO	0.5910	0.9256	0.6094	0.0221
LIO + Wheel	0.5726	0.9142	0.5887	0.0187
LIO + Wheel + Ground Filter	0.5334	0.8896	0.5517	0.0199
Full Method	0.4216	0.8074	0.4479	0.0228

**Table A2:** Global localization Absolute Trajectory Error (ATE) statistics of Run2 on the College Dataset.

Four Configurations	MAE (m)	Max Error (m)	RMSE (m)	STD (m)
LIO	0.6243	1.0690	0.6480	0.0301
LIO + Wheel	0.5864	0.9162	0.6039	0.0209
LIO + Wheel + Ground Filter	0.5016	0.8903	0.5198	0.0187
Full Method	0.4206	0.8157	0.4500	0.0256

**Table A3:** Global localization Absolute Trajectory Error (ATE) statistics of Run3 on the College Dataset.

Four Configurations	MAE (m)	Max Error (m)	RMSE (m)	STD (m)
LIO	0.5999	0.9647	0.6194	0.0238
LIO + Wheel	0.5777	0.9157	0.5941	0.0191
LIO + Wheel + Ground Filter	0.5329	0.8859	0.5516	0.0203
Full Method	0.4300	0.8168	0.4566	0.0235

**Table A4:** Global localization Absolute Trajectory Error (ATE) statistics of Run4 on the College Dataset.

Four Configurations	MAE (m)	Max Error (m)	RMSE (m)	STD (m)
LIO	0.5817	0.9252	0.5997	0.0213
LIO + Wheel	0.5851	0.9165	0.6022	0.0203
LIO + Wheel + Ground Filter	0.5263	0.8895	0.5450	0.0200
Full Method	0.4379	0.8104	0.4649	0.0244

**Table A5:** Global localization Absolute Trajectory Error (ATE) statistics of Run5 on the College Dataset.

Four Configurations	MAE (m)	Max Error (m)	RMSE (m)	STD (m)
LIO	0.5921	0.9236	0.6103	0.0219
LIO + Wheel	0.5795	0.9174	0.5961	0.0195
LIO + Wheel + Ground Filter	0.5302	0.8895	0.5496	0.0209
Full Method	0.4308	0.7992	0.4571	0.0234

**Table A6:** Global localization Absolute Trajectory Error (ATE) statistics of Run6 on the College Dataset.

Four Configurations	MAE (m)	Max Error (m)	RMSE (m)	STD (m)
LIO	0.5900	0.9198	0.6079	0.0214
LIO + Wheel	0.5793	0.9269	0.5968	0.0206
LIO + Wheel + Ground Filter	0.5030	0.8925	0.5196	0.0170
Full Method	0.4347	0.8091	0.4629	0.0253

**Table A7:** Global localization Absolute Trajectory Error (ATE) statistics of Run7 on the College Dataset.

Four Configurations	MAE (m)	Max Error (m)	RMSE (m)	STD (m)
LIO	0.5891	0.9314	0.6075	0.0220
LIO + Wheel	0.5756	0.9255	0.5929	0.0202
LIO + Wheel + Ground Filter	0.5124	0.8886	0.5292	0.0175
Full Method	0.4305	0.8137	0.4573	0.0238
